# Insulin resistance and its association with the components of the metabolic syndrome among obese children and adolescents

**DOI:** 10.1186/1471-2458-10-318

**Published:** 2010-06-07

**Authors:** Carlos Juárez-López, Miguel Klünder-Klünder, Patricia Medina-Bravo, Adrián Madrigal-Azcárate, Eliezer Mass-Díaz, Samuel Flores-Huerta

**Affiliations:** 1 Department for Innovation and Quality. Institute for Decentralized Public Health Services (INDESALUD), Campeche State, Mexico; 2 Community Health Research Department, Hospital Infantil de México Federico Gómez, Ministry of Health (SSA) Mexico City, Mexico; 3 Public Health Laboratory, Campeche State, Mexico

## Abstract

**Background:**

Insulin resistance is the primary metabolic disorder associated with obesity; yet little is known about its role as a determinant of the metabolic syndrome in obese children. The aim of this study is to assess the association between the degree of insulin resistance and the different components of the metabolic syndrome among obese children and adolescents.

**Methods:**

An analytical, cross-sectional and population-based study was performed in forty-four public primary schools in Campeche City, Mexico. A total of 466 obese children and adolescents between 11-13 years of age were recruited. Fasting glucose and insulin concentrations, high density lipoprotein cholesterol, triglycerides, waist circumference, systolic and diastolic blood pressures were measured; insulin resistance and metabolic syndrome were also evaluated.

**Results:**

Out of the total population studied, 69% presented low values of high density lipoprotein cholesterol, 49% suffered from abdominal obesity, 29% had hypertriglyceridemia, 8% presented high systolic and 13% high diastolic blood pressure, 4% showed impaired fasting glucose, 51% presented insulin resistance and 20% metabolic syndrome. In spite of being obese, 13% of the investigated population did not present any metabolic disorder. For each one of the components of the metabolic syndrome, when insulin resistance increased so did odds ratios as cardiometabolic risk factors.

**Conclusions:**

Regardless of age and gender an increased degree of insulin resistance is associated with a higher prevalence of disorders in each of the components of the metabolic syndrome and with a heightened risk of suffering metabolic syndrome among obese children and adolescents.

## Background

The World Health Organization recognizes overweight and obesity in children and adolescents as worldwide public health problems. Mexico is one of the countries which suffer most from these.

Indeed, according to Mexican National Health and Nutrition Surveys, in 1999 [1] the combined prevalence of overweight and obesity in school-age children was 18.6%; by 2006, such prevalence had increased to 26%, which represents an average increase of 1.1 percentage points per year [2].

Insulin resistance (IR) is the primary metabolic disorder associated with obesity and is defined as a diminished ability of insulin to stimulate glucose uptake by skeletal muscle and adipose tissue, in addition to reducing insulin's ability to suppress hepatic glucose production and output [3]. Some of the several disorders associated with IR that have been described include systemic inflammation, increases in fibrinolysis, endothelial dysfunction, and atherosclerosis [4], all of which can first appear during childhood in obese individuals [5]. Although the hyperinsulinemic-euglycemic clamp is considered the gold standard for evaluating and measuring IR, the technical difficulties associated with this method have led to the development of less invasive methods. Among these, the homeostasis model assessment of insulin resistance index (HOMA-IR) is one of the most commonly used [6]. It is worth noting that no consensus exists concerning the HOMA-IR cut-off points that define IR among the pediatric population, although there is general agreement that IR is a common pathway for the development of glucose metabolism disorders, dyslipidemias, and high blood pressure, all of which are components of the metabolic syndrome (MS) [7-9]. In turn, MS is a risk factor for the subsequent development of type-2 diabetes mellitus (T2DM) and cardiovascular disease (CVD) [10].

Among children, MS is not well characterized, and there is a lack of agreement as to its components and cut-off points, though most authors include among the former abdominal obesity, high blood pressure, glucose abnormalities, and dyslipidemias [11,12]. Due to this lack of consensus, the reported prevalence of MS among children and adolescents shows a great deal of variability [13,14]. For instance, in studies including children and adolescents with varying nutritional conditions, the prevalence lies between 2.5% and 12.9% [13]. However, when only overweight and obese children and adolescents are included, the prevalence increases and falls within the range 26% -31.2% [13-15].

In children and adolescents, a direct relationship between the degree of obesity and the prevalence of MS has been reported [16,17]; however, it is not exactly known how and to what extent IR is associated with each of the components of MS. In this context, this study aims to elucidate the prevalence of IR and to evaluate the association of IR with each of the components of MS among obese children and adolescents.

## Methods

The study was conducted in all of the forty-four schools that compose the public school system in Campeche City, Mexico.

Prior to the study, ethical clearance was obtained from Campeche State research ethics committees and school authorities. The study consisted of two stages, the first of which aimed to identify children suffering from obesity. For this purpose, weight and height were measured by four nurses previously trained to follow international anthropometric guidelines. The study included 4,937 children between 11 and 13 years of age attending the 5^th ^and 6^th ^grades. Obesity was defined as a body mass index (BMI) percentile ≥95^th ^for the child's age and gender according to the Centers for Disease Control (CDC) 2000 references [18]. Following this criterion, 1,475 children were classified as obese. Out of this total, written parental and child informed consent was obtained from 600 randomly selected children. From this group, complete blood samples were collected from 466 children.

During the second stage, blood pressure (BP) was obtained using the auscultatory method, and waist circumference (WC) was measured with children in the standing position, placing the metric strip at the midpoint between the lower rib and the iliac crest after a normal exhalation. In addition, a sample of venous blood was obtained after a 12-hour fast in order to determine concentrations of insulin by chemiluminescence immunoassay (Access Beckman Coulter Instruments, Brea California), glucose, and plasma lipids by an enzymatic method, and high density lipoprotein cholesterol (HDL-C) assayed by the addition of magnesium ions (Synchron CX^® ^Beckman Instruments, Brea California). In all cases, commercial enzymatic kits were employed. Low density lipoprotein cholesterol (LDL-C) levels were estimated using the Friedewald formula, as modified by De Long [19].

### Definition of MS and its components

MS was defined according to guidelines by the International Diabetes Federation (IDF) [12], the only exception being that the BP criteria were used according to the North American Task Force guidelines [20]. For each of the components of MS, the following cut-off points were used: Hypertriglyceridemia (triglycerides ≥ 150 mg/dL), low HDL-C (HDL-C ≤ 40 mg/dL), high blood pressure (systolic and/or diastolic BP ≥ 90^th ^percentile for children's age, gender, and height), impaired fasting glucose (fasting glucose ≥ 100 mg/dL), and abdominal obesity (WC ≥ percentile 90^th ^for children's age, gender, and ethnic origin) [21]. MS was diagnosed when three or more of the previously described components were present. Hypercholesterolemia (total cholesterol ≥ 200 mg/dL) and high values of LDL-C (LDL-C ≥ 130 mg/dL) were defined according by the American Academy of Pediatrics [22].

### Definition of IR

IR was determined through HOMA-IR, which was calculated using the following equation: [(fasting glucose (mg/dL))(fasting insulin (μU/mL))/405)] [23]. A HOMA-IR value of 3.4 was chosen as the cut-off point to define IR as it has been suggested that beyond this value, which corresponds to the 90^th ^percentile of a population of healthy children, IR becomes a cardiovascular risk factor [24].

#### Statistical analysis

Means and standard deviations and prevalence of anthropometric and metabolic variables were obtained. These measures were compared by gender using Student's t test or X2 test. Four categories were derived for HOMA-IR percentiles: <25^th^, 25-49.9^th^, 50-74.9^th^, and ≥75^th^. In order to assess the risk of presenting disorders in each of the MS components, measures of these were compared according to the aforementioned HOMA-IR percentile categories through logistic regression analysis. Statistically significant differences were assumed if the *P*-value was < 0.05.

Data were processed with STATA, SE v.9.0, and EPIINFO 3.3.2 according to the CDC 2000 reference [18].

## Results

Table 1 depicts the anthropometric features, BP and metabolic profile of the obese children and adolescents who participated in the study. For both sexes, the mean age was 11.3, SD 0.8 years; the mean BMI, 27.3 kg/m², SD 3.0, which corresponds to the 97.5^th ^percentile for age and gender; and the mean WC, 85.0 cm, SD 7.8.

**Table 1:** Anthropometric measures, blood pressure and metabolic profile of obese children and adolescents

Features	Total n = 466	Boys n = 272 (58.4%)	Girls n = 194(41.6%)	P*
Age (years)	11.3 (0.8)	11.3 (0.8)	11.3 (0.8)	0.629
**Anthropometric Measures**				
Weight (Kg)	59.7 (10.0)	59.0 (10.2)	60.7 (9.6)	0.075
Height (m)	1.5 (0.7)	1.5 (0.7)	1.5 (0.6)	0.119
Body mass index (kg/m²)	27.3 (3.0)	27.1 (3.0)	27.5 (2.9)	0.164
Body Mass Index Percentile	97.5 (1.2)	97.6 (1.2)	97.3 (1.3)	0.010
Waist circumference (cm)	85.0 (7.8)	86.2 (8.1)	83.4 (7.1)	<0.001
**Blood Pressure (mmHg)**				
Systolic	105.7 (10.1)	106.2 (10.0)	105.0 (10.2)	0.189
Diastolic	66.2 (8.6)	66.3 (8.9)	66.1 (8.3)	0.822
**Metabolic profile**				
Glucose (mg/dL)	88.7 (6.6)	89.2 (6.7)	87.9 (6.5)	0.033
Insulin (μU/mL)	18.8 (12.1)	16.9 (11.3)	21.5 (12.7)	<0.001
HOMA-IR^&^	4.2 (2.9)	3.8 (2.8)	4.7 (2.9)	<0.001
Total cholesterol (mg/dL)	166.1 (30.2)	169.2 (32.5)	161.9 (26.1)	0.010
Triglycerides (mg/dL)	135.0 (75.3)	132.9 (77.1)	137.8 (72.8)	0.489
LDL-C (mg/dL)	101.2 (25.5)	104.3 (27.7)	96.9 (21.3)	0.002
HDL-C (mg/dL)	38.0 (8.2)	38.3 (8.4)	37.5 (7.9)	0.267

All values are means (standard deviations).

* *P *value according to Student's *t *test comparing boys and girls.

^&^HOMA-IR: [(fasting glucose (mg/dL))(fasting insulin (μU/mL))/405)]

No difference was observed between sexes in the values of systolic and diastolic BP. However, in both sexes systolic BP fell into the 49.5^th ^percentile, whereas diastolic BP fell into the 60.9^th ^percentile, according to the reference values for gender, age, and height [20] (these data are not shown in the tables).

The same table summarizes the values of glucose, insulin, and lipids in the investigated children and adolescent population. Girls exhibited a higher concentration of fasting insulin and higher HOMA-IR values compared to boys, but smaller concentrations of fasting glucose, total cholesterol, and LDL-C.

Table 2 depicts the prevalence of cardiometabolic risk factors and the components of MS among the study participants. The prevalence of the components of MS in this population was as follows: abdominal obesity, 49%; high systolic BP, 8%; high diastolic BP, 13%; impaired fasting glucose, 4%; hypertriglyceridemia, 29%; and low HDL-C, 69%. Although 56% of the population suffered from hyperinsulinemia, this condition was observed in 71% of girls but only in 45% of boys (*P *< 0.001); likewise, IR was more common among girls than boys, 63% versus 43%, respectively (*P *< 0.001). The prevalence of heightened total cholesterol levels was higher among boys (14%) than girls (7%) (*P *= 0.013), a similar pattern also found in relation to increased LDL-C values, where the prevalence was 15% and 6% (*P *= 0.004) among boys and girls, respectively. Although 13% of these children and adolescents did not have any components associated with MS, 20% had three or more of such components.

**Table 2:** Prevalence of cardiometabolic risk factors in obese children and adolescents

	Total (n = 466)	Boys (n = 272)	Girls (n = 194)	P*
Waist circumference ≥ 90 pc ^a^	49	48	51	0.491
Systolic BP ≥ 90 pc ^b^	8	10	7	0.272
Diastolic BP ≥ 90 pc ^b^	13	14	11	0.579
Glucose ≥ 100 mg/dL ^c^	4	6	2	0.063
Insulin ≥ 15 μU/mL ^d^	56	45	71	<0.001
HOMA-IR (≥ 3.4) ^d^	51	43	63	<0.001
Total cholesterol ≥ 200 mg/dL ^e^	11	14	7	0.013
Triglycerides ≥ 150 mg/dL ^c^	29	28	31	0.431
LDL-C ≥ 130 mg/dL ^e^	11	15	6	0.004
HDL-C < 40 mg/dL ^c^	69	68	71	0.495
Metabolic syndrome components ^c^				
0	13	15	10	0.162
1	30	28	31	0.465
2	37	36	38	0.700
≥3 components ^c^	20	21	20	0.898

All values are percentages.

^a ^According to Waist Circumference percentiles of Mexican-American children [21].

^b ^According to National High Blood Pressure Education Program [20].

^c ^According to International Diabetes Federation (IDF) criteria [12].

^d ^Insulin and HOMA-IR According to the cardiovascular risk cut-off points [24].

^e ^According to Committee on Nutrition of the American Academy of Pediatrics [22].

*P value according to *X*² test comparing boys and girls.

Using the group with HOMA-IR values below the 25^th ^percentile as a reference, table 3 shows how, for each one of the components of MS, when IR increases so does the "odds ratio (OR)" as a cardiometabolic risk factor. WC begins to be significant from the 25^th ^percentile of IR (*P *= 0.014). From the 50^th ^percentile onwards, in addition to WC, also diastolic BP, glucose and triglycerides become significant (*P *< 0.05). Above the 75^th ^percentile, all of the OR are significant (*P *< 0.05), except systolic and diastolic BP.

**Table 3:** Odds ratios of suffering cardiometabolic risk factors according to HOMA-IR percentiles in obese children and adolescents

Cardiometabolic risk factors	HOMA-IR Percentile (values)			
	
	<25 (<2.4)	25-49.9 (2.4-3.3)	50-74.9 (3.4-4.9)	≥75 (≥5.0)
WC^a ^≥90 pc				
OR	Referent	2.0	3.3	5.2
95% CI	-	1.2; 3.5	1.9; 5.8	2.9; 9.3
*P*	-	0.014	<0.001	<0.001
Systolic BPb ≥ 90 pc				
OR	Referent	0.5	2.0	2.2
95% CI	-	0.2; 1.9	0.8; 5.0	0.9; 5.6
*P*	-	0.326	0.159	0.098
Diastolic BPb ≥ 90 pc				
OR	Referent	1.5	2.5	1.8
95% CI	-	0.6; 3.5	1.1; 5.8	0.8; 4.3
*P*	-	0.395	0.025	0.161
Glucose ≥ 100 mg/dL				
OR	Referent	1.9	4.9	5.0
95% CI	-	0.3; 12.0	1.0; 24.7	1.0; 25.3
*P*	-	0.475	0.052	0.050
Triglycerides ≥ 150 mg/dL				
OR	Referent	1.8	2.8	4.3
95% CI	-	0.9; 3.5	1.5; 5.4	2.3; 8.2
*P*	-	0.083	0.002	<0.001
HDL-C < 40 mg/dL				
OR	Referent	1.1	1.4	1.8
95% CI	-	0.6; 1.9	0.8; 2.5	1.0; 3.2
*P*	-	0.714	0.237	0.050

Values obtained through logistic regression, adjusted for gender and age

^a ^WC: Waist circumference ^b ^BP: Blood pressure

Likewise, Table 4 shows the ORs of suffering MS according to IR categories. From this table, it is apparent that the Odds of developing MS (adjusted for gender and age) increases as a function of IR. Such OR is 5.5 (95% CI 2.6-11.6) times greater when IR is above the 75^th ^percentile.

**Table 4:** Risk of suffering from metabolic syndrome according to HOMA-IR* percentile values

HOMA-IR Percentile (values)	OR	95% CI	*P*
<25 pc (<2.4)	Referent	-	-
25-49.9 pc (2.4-3.3)	1.3	(0.5; 2.9)	0.602
50-74.9 pc (3.4-4.9)	3.9	(1.8; 8.2)	0.001
≥75 pc (≥5.0)	5.5	(2.6; 11.6)	<0.001

*Values obtained through logistic regression analysis, adjusted for gender and age

## Discussion

Our results show that higher levels of IR are associated with a greater degree of alterations in the components of the MS in the population studied, half of which presented IR.

The apparently normal fasting glucose levels in this population are maintained by a compensatory mechanism based on hyperinsulinism, which is reflected in HOMA-IR values [25]. However, when impaired fasting glucose prevalence is analyzed through ORs, it is possible to observe that increased levels of IR are associated with rising OR.

Girls presented the highest insulin levels and HOMA-IR values, along with lower glucose concentrations. This pattern could be a consequence of the fact that, at equal ages, girls can enter puberty up to two years earlier than boys [26,27] - therefore, more girls would have reached higher pubertal stages. However - and this is a limitation of this study - no information was collected about either pubertal stage or growth and sexual hormones, factors which could influence the prevalence of the rise in IR [28].

In accordance with previous studies on adolescents and adults, the IR reported in this study is associated with the primary alterations in the lipid profile; hyperinsulinism increases the free fatty acid release and the triglyceride synthesis, which results in hypertriglyceridemia [29,30]. Likewise increased hepatic lipase activity can account for the rise in high-density lipoprotein depuration, producing hypoalphalipoproteinemia [31]. However, the high prevalence of low C-HDL levels observed in this study is one of the highest reported in the literature [13,30,32]. Regardless of its cause, it is a risk factor for the development of cardiovascular events during adulthood. Such events stem from both genetic and environmental factors. Indeed, it has been reported that individuals of Turkish descent display greater hepatic lipase activity, which augments the depuration of this lipoprotein and is associated with lower levels of HDL-C [33]. However, it is not known whether population of Mexican descent could have a polymorphism such as that identified among their Turkish counterparts that may explain the higher prevalence of low HDL-C observed in this study. What is known is that, among individuals of Mexican descent, an association has been described between a variant of gene ABCA1 and lower HDL-C levels, along with a greater risk of developing obesity, MS, and early onset T2DM [34]. As far as environmental factors are concerned, lower levels of this lipoprotein can be explained in terms of changing eating habits [35]. The diet of Mexican children resembles ever more that of their North American counterparts [36], a diet rich in simple sugars and animal fats, but with limited amounts of fiber [37].

Although the association between IR and OR of suffering high blood pressure was not statistically significant, the observed trend in this population can be associated with the different disorders stemming from obesity [7] among which IR stands out [38]. Hyperinsulinism increases renal sodium absorption and sympathetic tone [39], which combines with altered vasodilatation, which in turn is a secondary effect of nitric oxide deficient secretion by the vascular endothelium [40]. Therefore, it is to be expected that if such IR-induced hyperinsulinism continues, permanent alterations such as atherosclerosis and hypertension will eventually develop.

Furthermore, the prevalence of MS reported in this study - around 20% - might stem from the employed definition, which relied on stricter cut-off points than those used in other studies [11]. In studies where Cook's criteria was applied, the prevalence of MS among North American obese adolescents was 27% [16], in others following the III Adult Treatment Panel criteria, the prevalence was 26.1% [15].

In order to ascertain the real prevalence of this syndrome among children, agreement is needed concerning the cut-off points for each of the syndrome's components; likewise, categories for pubertal stage and gender need to be established [41]. Notwithstanding the heterogeneity of current definitions, the notion of MS has been useful for identifying individual children at risk of developing CVD and T2DM [42].

As mentioned above, 87% of the study participants presented at least some kind of functional or metabolic disorder. Nevertheless, in spite of being obese, almost 13% of the study population did not present any kind of disorder, a phenotype known as "metabolically healthy but obese individuals" [43]. Among adults, a 9.7% prevalence of this phenotype has been reported in the literature; interestingly, within this group no family history of T2DM has been found [44]. This suggests that protection against cardiometabolic disorders, even in the presence of obesity, can have genetic features [45].

A further limitation of this study derives from the fact that cross-sectional studies are not able to establish causality, only associations between different variables.

However, given the close relationship between obesity, IR, and MS - with its attendant risk of developing co-morbidities, such as T2DM and CVD - it is imperative to implement prevention, diagnosis, and early treatment measures for obesity involving all sectors of society.

## Conclusions

This study has confirmed that among obese children and adolescents, regardless of age and gender, an increased degree of insulin resistance is associated with a higher prevalence of disorders in each of the components of the metabolic syndrome and with a heightened risk of suffering MS.

HOMA-IR values above 3.4, which correspond to the 50^th ^percentile of this population, were associated with an increased risk of having MS, compared to the lowest percentile of HOMA-IR values.

## Competing interests

The authors declare that they have no competing interests.

## Authors' contributions

CJL conceived of the study, and participated in its design and coordination. MKK and PMB performed the statistical analysis and revised the manuscript critically. AMA participated in administrative and technical support, obtained data and supervised the work field. EMD provided administrative and technical support, obtained data and analysed blood samples. SFH participated in the conception and design of the research question as well as in fieldwork supervision. All authors were involved in drafting the manuscript. All of them read and approved the final version of the manuscript.

## Pre-publication history

The pre-publication history for this paper can be accessed here:

http://www.biomedcentral.com/1471-2458/10/318/prepub
